# The Use of miRNA Panel as a Growth Plate Marker of Short‐Term Response to GH

**DOI:** 10.1111/cen.15278

**Published:** 2025-05-20

**Authors:** Caroline Rosa Pellicciari, Thayna Rosa Bispo, Itatiana Ferreira Rodart, Leandra Steinmetz, Adriana Aparecida Siviero Miachon, Angela Maria Spinola e Castro, Durval Damiani, Cristiane Kochi, Carlos Alberto Longui

**Affiliations:** ^1^ Molecular Medicine Laboratory, Physiology Department Santa Casa de São Paulo School of Medical Sciences Sao Paulo SP Brazil; ^2^ Pediatric Endocrinology Unit, ICr‐USP Sao Paulo University Medical School Sao Paulo SP Brazil; ^3^ Pediatric Endocrinology Unit Federal University of Sao Paulo, UNIFESP Sao Paulo SP Brazil; ^4^ Pediatric Endocrinology Unit, Pediatrics Department Irmandade da Santa Casa de Misericórdia de São Paulo Sao Paulo SP Brazil

**Keywords:** Digital PCR (dPCR), Growth hormone, Growth hormone deficiency, MicroRNAs (miRNAs)

## Abstract

**Introduction:**

Recombinant human growth hormone (GH) therapy shows variable growth responses in patients with growth hormone deficiency (GHD), highlighting the need for reliable biomarkers to predict individual sensitivity.

**Objective:**

This study investigated circulating microRNAs (miRNAs) involved in growth plate regulation during GH therapy in prepubertal children with GHD, aiming to establish a miRNA panel correlating with GH responsiveness.

**Patients and Methods:**

Sixteen patients were treated with daily recombinant GH (0.033 mg/kg) for 6 months. Two participants were excluded for protocol noncompliance, and one withdrew due to the spontaneous onset of puberty. Circulating levels of six miRNAs (miR‐22‐3p, miR‐30c‐5p, miR‐140‐5p, miR‐340‐5p, miR‐494‐3p, and miR‐16‐5p) were measured via digital PCR at baseline and at 1, 3, and 6 months of therapy. Clinical data (height, weight, growth velocity) and hormone levels were collected simultaneously.

**Results:**

All miRNAs displayed a characteristic response pattern: significant increases at 1 and 6 months, with a transient decline at 3 months. Despite these changes, no significant correlations were identified between miRNA levels and growth velocity, height SDS, or IGF‐1 levels. The study included treatment‐naïve patients and those with prior GH therapy following a 30‐day wash‐out period. While the treatment‐naïve group exhibited greater height‐SDS gains (*p* = 0.008), no differences in miRNA expression were observed between groups, nor was there a correlation between miRNA levels and height‐SDS increments.

**Conclusion:**

While this miRNA panel identified treatment‐responsive miRNAs, it did not correlate with growth outcomes. Increasing the sample size and incorporating additional miRNAs could enhance its clinical utility for predicting GH treatment sensitivity in GHD patients.

**Trial Registration:**

NCT05946915.

## 1 Introduction

The clinical experience with the therapeutic use of recombinant human growth hormone (GH) has found to be adequate, but with variable amplitude of growth response [1]. Poor short‐term response has been translated into an unsatisfactory gain in adult height [2]. Mathematical models were developed with the aim to improve the prediction accuracy of growth and final height, but can only explain ~60% of the GH induced height increment observed GH deficiency patients (GHD) [2, 3]. Biochemical variables such as baseline IGF‐1 and leptin have been added to the prediction models with mild impact [3]. Genetic markers such as exon 3‐deleted growth hormone receptor polymorphism and IGFBP3 polymorphisms were also used in an attempt to discriminate good and bad responders to GH treatment, but the results were contradictory in different studies [4, 5].

MicroRNAs (miRNAs) are small noncoding RNAs that regulate gene expression. Most genes in the human genome are regulated by one or more miRNAs [6]. MicroRNAs appear to play an important role in the development of physiological processes, as well as in human diseases [7], usually suppressing gene expression by binding to the 3'UTR domain of mRNA [8], therefore increasing mRNA degradation or preventing translational information to protein synthesis. One of the remarkable characteristics of miRNAs is its plasma circulation inside exosomes, conferring resistance to degradation, and allowing its measurement in the circulating blood, also known as a liquid biopsy [9].

In vitro models and animal studies suggested that miRNAs have an important role in the regulation of endochondral ossification and also in the regulation of the hypothalamic‐pituitary‐IGF axis [10]. In particular, miRNA‐140, miRNA‐322 and miRNA‐22 have been described as involved in growth plate development [11]. Several other miRNAs, such as miR‐22‐3p, miR‐30c‐5p, miR‐106a‐5p, miR‐140‐5p, miR‐199a‐5p, miR‐335‐5p, miR‐340‐5p, and miR‐494‐3p are also involved in the regulation of longitudinal growth and bone development, through its action upon WNT‐βcatenin, Notch, PI3K/AKT and TGFβ signaling pathways [10].

**Figure 1:**
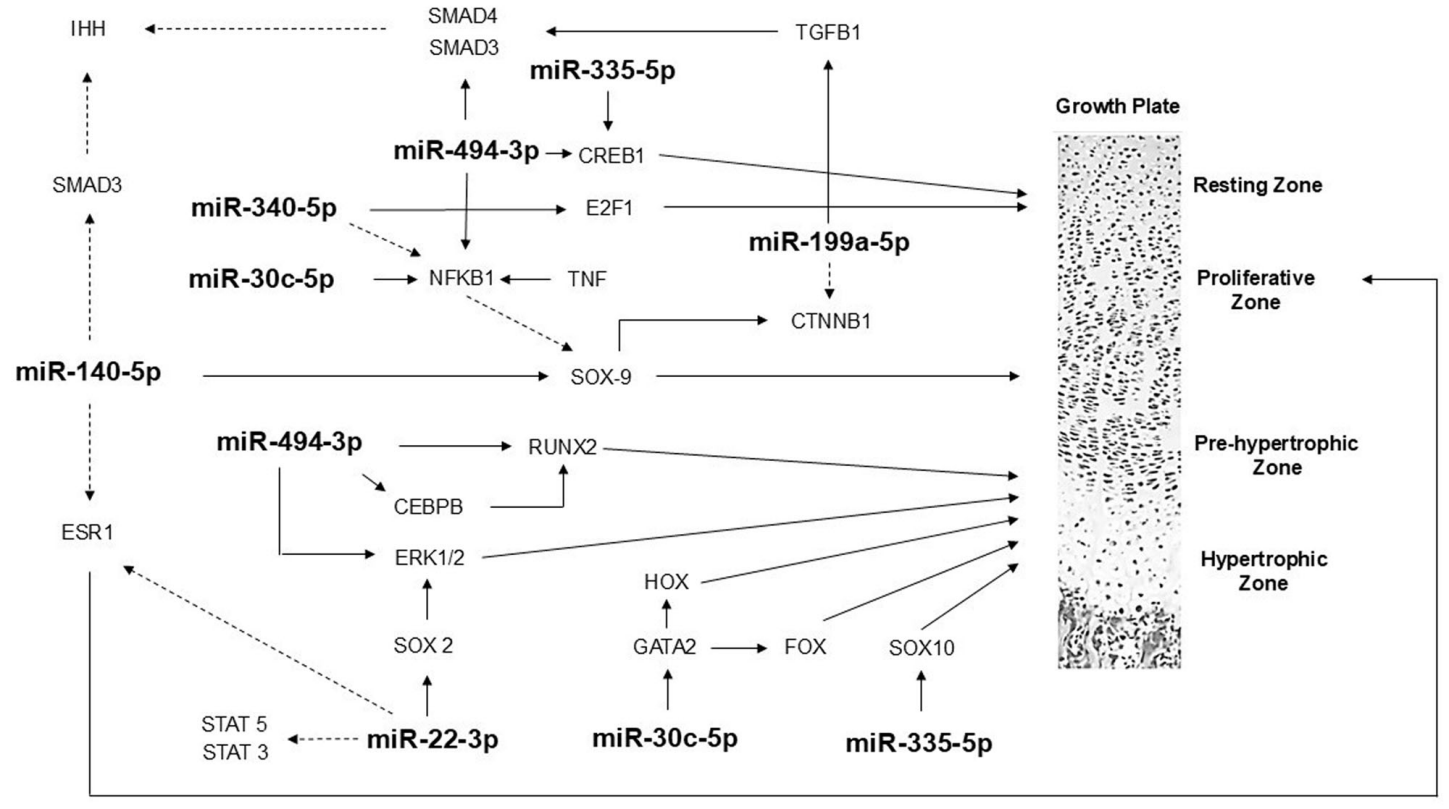
Schematic representation of the main interactions of microRNAs in the growth plate (employing the site TransmiR v2.0 database at: http://www.cuilab.cn/transmir.

By elucidating the key mechanisms involved in activation‐differentiation‐apoptosis of growth plate chondrocytes it is now possible to understand the importance of microRNAs in the entire process (Figure 1).

Growth response in patients during GH treatment is variable, depending both on the patient's basal conditions and on individual innate sensitivity to therapy [12]. Expected and measured growth rate can be discordant, and the degree of correlation between clinical–auxological parameters, GH dose and GH peak response during stimulation tests vary enormously, both inter and intra‐individuals during treatment [13].

Therefore, the aim of this study was to measure the circulating growth plate interfering miRNAs before and during GH treatment, eventually establishing a panel of miRNAs that could correlates with GH responsiveness. This miRNA panel would have a huge clinical applicability, allowing the identification of patients who need differential therapeutic protocols targeting the achievement of the best response during GH treatment.

## 2 Materials and Methods

This is a prospective interventional clinical trial (**ClinicalTrials.govID** NCT05946915) with primary data analysis carried out from April 2023 to October 2024. It was approved by the research ethics committee of our institution, under approval number ‐ CAAE: 67005222.0.0000.5479. All patients attended the pediatric endocrinology outpatient clinic of Santa Casa Hospital (philanthropic institution) in São Paulo, Brazil.

A total of 16 prepubertal children with growth hormone deficiency were screened to participate in the study. Two patients were excluded for do not follow the protocol properly and one patient was excluded due to spontaneous onset of puberty. Therefore, 13 patients were included in the final analyses (11 males, 84.6%).

At the time of diagnosis, all subjects underwent two GH stimulation tests (clonidine and insulin or glucagon tests) showing a GH‐peak concentration of less than 5 ng/ml. All patients had performed a magnetic resonance imaging scan of the hypothalamus and pituitary gland, recognizing in 12 patients (92.3%) the presence of Posterior Pituitary Ectopic gland. Only one case was considered as idiopathic GH deficiency.

Patients were screened for other hormone deficiencies, and appropriate hormone replacements were administered before and during the study period when necessary. Nine patients were diagnosed with ACTH deficiency and received glucocorticoid replacement therapy. Prednisolone was selected due to its convenient once‐daily dosing schedule, with an average dose of 1.3 mg/m²/day, adjusted according to each patient's weight over the course of the study. In addition, seven patients had TSH deficiency and were treated with daily sodium levothyroxine. Dosages were adjusted, when necessary, based on serum free T4 (fT4) levels.

The exclusion criteria included patients with chronic diseases, those requiring prolonged supraphysiologic doses of glucocorticoids, bone age greater than 10 years, and patients lacking adequate follow‐up during the study protocol. GH treatment was done in accordance to the guidelines of the Brazilian Regulatory Agency. The same recombinant biosimilar human GH were employed to treat all patients at a daily dose of 0.033 mg/kg.

Clinical records at each visit included age, weight, height, BMI, height‐to‐target height (TH) difference, and pubertal stage. Clinical evaluations were scheduled to be conducted at four time points: before starting medical therapy (baseline), and at 1, 3, and 6 months during GH treatment. Height, target height and BMI were expressed as SD scores (SDS) according to the WHO 2007 standards. Growth velocity SDS was calculated using Tanner references [14]. The annualized growth velocity was determined through a prospective evaluation of height over a 6‐month period, with all measurements performed by the same investigator, the pediatric endocrinologist (CRP), to ensure consistency and reduce measurement variability. Puberty in all subjects was classified as stage I during the whole period of treatment, according to Marshall and Tanner's criteria [15, 16]. Bone age was assessed every 6 months and blinded rated by the same observer according to the Greulich and Pyle method [17].

Laboratory Assessment: Hormone levels were measured at baseline, and at 1, 3, and 6 months, including IGF‐1, fasting glucose, insulin, TSH, free T4, and cortisol. Gonadotropins and sex steroids were measured when appropriate to reaffirm the prepubertal stage.

To evaluate the expression of growth plate related miRNAs, a baseline blood sample was obtained either before starting GH therapy or after a short time washout period (30 days), and followed by additional samples at 1, 3, and 6 months during GH treatment.

MiRNAs determination: Quantitative PCR (digital PCR—dPCR) was employed to measure circulating miRNA levels. This technique is considered the gold standard in liquid biopsy applications due to its superior precision and sensitivity. Unlike traditional PCR, dPCR is less affected by inhibitors and does not require internal or external normalization when detecting low concentrations of target nucleic acids. Briefly, the whole blood samples were drawn into vacutainer serum separator tubes and processed within 2 h of collection. The samples were centrifuged at 2000 *g* for 10 min at 4°C. Then, the serum was aliquoted into 1.5 ml sterile RNase‐free tubes and further centrifuged at 2,500 g for 10 min at 4°C to remove any contaminant cells and debris. The purified serum was collected in sterile RNase‐free tubes and stored at −80°C until use. Total RNA was isolated from 200 μl of serum using the miRNeasy Serum/Plasma Advanced Kit (ID: 217204 ‐ Qiagen) and subsequently converted to cDNA. RNA extraction as well as cDNA conversion were performed for each patient at the same time point, minimizing the possibility that technical failures during extraction or conversion. The following miRNAs were measured: hsa‐miR‐22‐3p, hsa‐miR‐30c‐5p, hsa‐miR‐140‐5p, hsa‐miR‐340‐5p, hsa‐miR‐494‐3p and hsa‐miR‐16‐5p. Each cDNA sample was diluted 1:30. For the hsa‐miR‐22‐3p and hsa‐miR‐30c‐5p dPCR assays, the reaction mixture consisted of: 10 μL cDNA sample, 13.3 μL 3× Evagreen PCR Master Mix (Qiagen), 4 μL Primer Mix (Qiagen) and 12.7 μL of RNase‐free water, resulting in a 40 μL total reaction volume. For the hsa‐miR‐140‐5p, hsa‐miR‐340‐5p, and hsa‐miR‐494‐3p dPCR assays, the reaction mixture consisted of 22.7 μL of cDNA sample, 13.3 μL of 3x Evagreen PCR Master Mix (Qiagen), and 4 μL of Primer Mix (Qiagen), also resulting in a 40 μL total reaction volume. For the hsa‐miR‐16‐5p dPCR assays, the reaction mixture consisted of 5 μL of cDNA sample, 13.3 μL of 3× Evagreen PCR Master Mix (Qiagen), and 1 μL of Primer Mix (Qiagen), and 20.7 μL of RNase‐free water, also resulting in a 40 μL total reaction volume. The dPCR assay mixture was then pipetted into a QIAcuity™ nanoplate 26k 24‐well and inserted into the QIAcuity One digital PCR system, where the DNA template was distributed randomly. The cycling conditions were set as follows: 95°C for 2 min, followed by 40 cycles of 95°C for 15 s and 56°C for 1 min, with a final step at 40°C for 5 min. Exposures were set to 250 ms with a gain of 3. The detection of the amplification target at the end of the PCR process was achieved by examining the presence or absence of fluorescence, which results from the use of intercalating dyes. Due to the random partitioning of the PCR reactions, it is possible for a positive reaction to contain multiple target molecules. To account for this, the Poisson model is employed to calculate the likelihood of a micro reaction containing zero, one, two, three, four, or five copies of the target molecule. The total number of target copies in all valid partitions of a well is determined by multiplying the average number of target molecules per partition by the total number of valid partitions. Additionally, the concentration in copies per microliter can be calculated based on the known number of target copies per partition and the partition volume. All data analyses were conducted using the QIAcuity Suite software (Qiagen, Hilden, Germany).

Statistical Analysis: For statistical analysis, the SigmaStat 3.5 software (Systat Software Inc) was employed. Clinical variables were expressed as SDS for sex and age. Laboratory variables were described in terms of central tendency values such as mean (SD) and median (p25‐p75) at different time‐points. The comparison of the same variable during treatment applied the ANOVA for repeated measures on Rank test (considering the small sample size and the nonparametric distribution). The association between two variables was analyzed by the Spearman Rank Order test and by regression analysis when appropriate. A 95% confidence interval was used, with *p* < 0.05 considered as statistically significant.

## 3 Results

**Figure 2:**
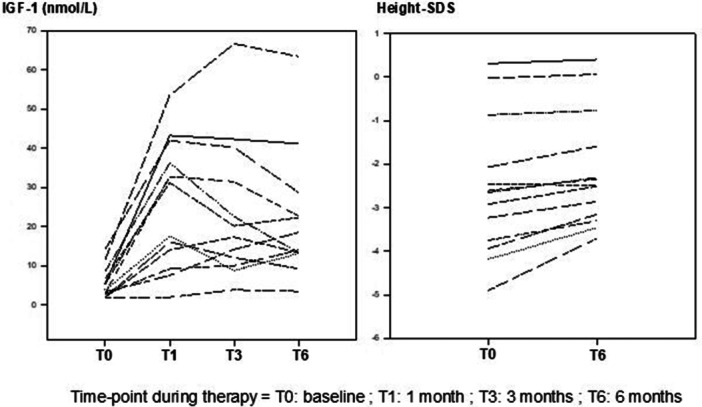
Longitudinal response curves of individual IGF‐1 levels, and height SDS throughout the treatment period.

The clinical and biochemical characteristics of the 13 subjects in the different time point of the study are summarized in Table 1. There was a significant improvement of 0.5 SD in 6 months of GH treatment, corresponding to mean annual growth velocity of 9.8 cm/year. IGF‐1, fasting glucose and insulin also increased significantly. To illustrate the longitudinal changes observed throughout the treatment period, we have included Figure 2, which presents individual response curves for IGF‐1 concentrations and height SDS.

**Table 1:** Auxological and biochemical features of 13 patients with GHD at baseline, 1, 3 and 6 months of GH treatment (0.033 mg/kg/day).

	Baseline	1 month	3 months	6 months
Gender M/F	11/02	11/02	11/02	11/02
CA, years	8.0 (3.5)	8.1 (3.5)	8.1 (3.6)	8.6 (3.6)
Midparental height, SDS	−0,7 (0.9)			
GH peak, ng/ml	1.15 (0.9)			
Bone age, years	6.5 (3.3)			7.2 (3.2)
Height, cm	112.5 (22.5)	113.6 (22.3)	113.7 (22.0)	117.6 (21.7)
Height, SDS	−2.6 (1.6)	−2.5 (1.5)	−2.5 (1.4)	−2.1 (1.3)
Weight, kg	24.2 (13.9)	26.1 (15.2)	26.2 (15.7)	27.2 (14.3)
BMI, Kg/m²	17.2 (3.6)	18.5 (4.0)	17.5 (2.8)	18.3 (3.6)
BMI, SDS	0.4 (1.4)	0.9 (1.3)	0.8 (1.3)	0.7 (1.4)
GV, cm/year				8.7 (2.5)
Predicted GV, cm/year				10.0 (3.1)
IGF‐1, nmol/L	5.41 (3.87)	25.96 (15.90)	24.09 (18.14)	20.62 (12.99)
Fasting glucose, mg/dl	73.4 (6.6)	81.1 (8.6)	81.2 (9.5)	81.2 (7.4)
Insulin, μU/ml	3.2 (2.3)	8.1 (5.2)	7.8 (7.7)	7.7 (8.5)
TSH, μU/ml	1.1 (1.5)	1.2 (1.5)	1.5 (1.9)	1.8 (2.1)
Free T4, ng/dl	1.2 (0.1)	1.0 (0.2)	1.0 (0.2)	1.1 (0.2)

*Note:* CA chronological age; GH peak after stimulation test (clonidine or insulin). GV growth velocity.

Predicted GV was calculated according to the respective year of GH treatment, as outlined in the KIGS‐based approach [3].

Reference ranges: IGF‐1: 2.35–41.40 nmol/L; Fasting glucose: 70–90 ng/dl; Insulin: 3.0–25.0 μU/ml; TSH: 0.3‐6.0 μU/ml; Free T4: 0.81–1.76 ng/dl.

**Figure 3:**
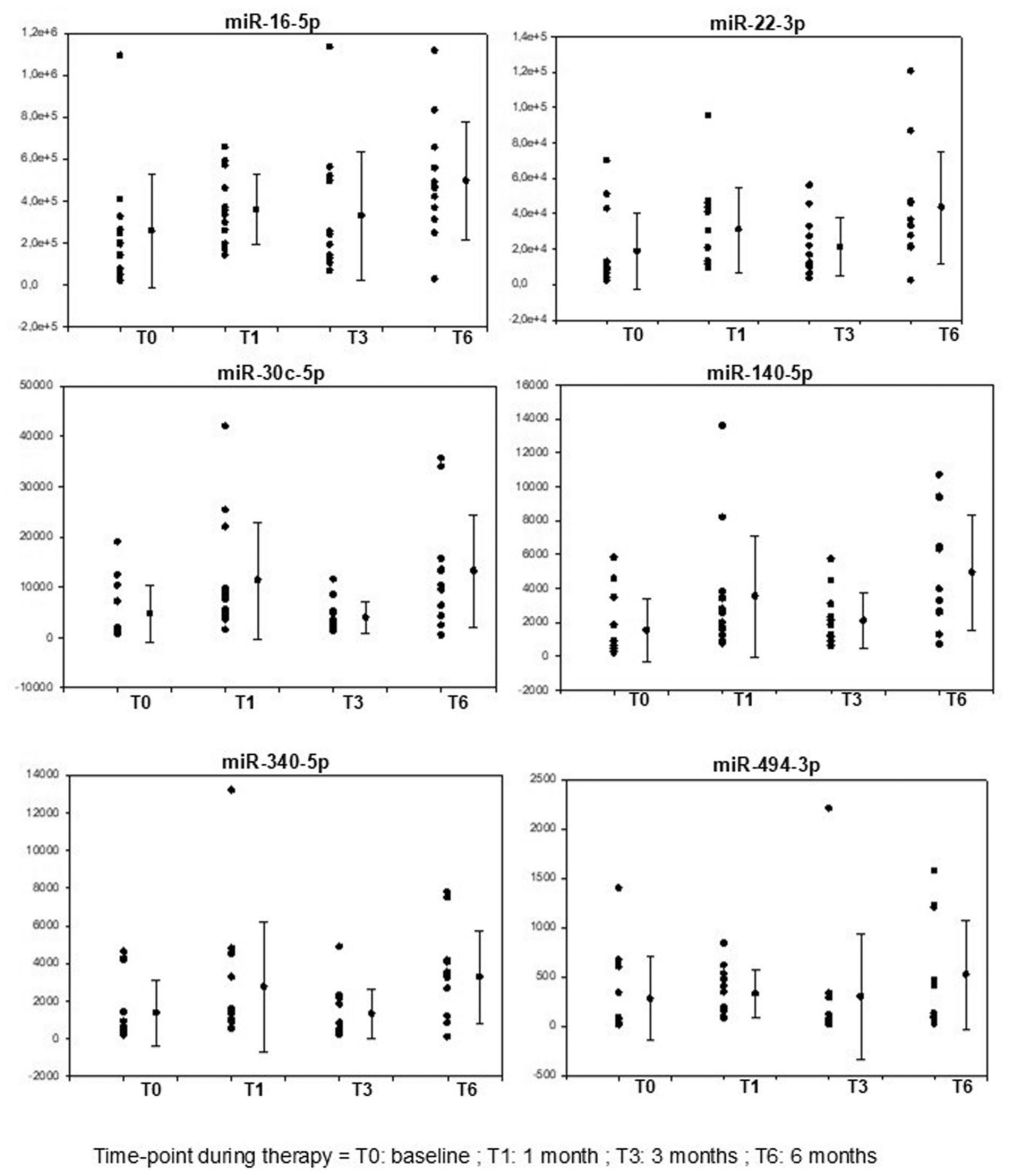
Individual results of each microRNA copy number measured by digital PCR at basal condition and after 1, 3 and 6 months of GH therapy. A pattern of significant increment of miRNA concentration at 1‐month, relative reduction at 3‐month and subsequent maximal peak at 6‐month was identified.

We could recognize a characteristic pattern of response to GH in all miRNA from the panel studied (Figure 3), with an initial increment after 1 month, followed by decrease at the 3‐month, with subsequent increase at the 6‐month timepoint. In our study, we included both treatment‐naïve patients and those with prior GH therapy in which a wash‐out period of 30 days were done before basal sample collection. Initially, we analyzed patients divided into two groups: a wash‐out group (30 days without GH use) and a no wash‐out group (treatment‐naïve). The miRNA concentration in the total cohort, wash‐out group, and treatment‐naïve group from basal condition to each timepoint of the study (1, 3, and 6 months) is described in Table 2.

**Table 2:** Median copy number of circulating microRNAs in the total cohort, wash‐out group, and treatment‐naïve group: comparison across baseline, 1, 3, and 6 months of GH therapy.

	Basal			1 month			3 months			6 months		
miRNA	Total	Wash‐out group	Naïve group	Total	Wash‐out group	Naïve group	Total	Wash‐out group	Naïve group	Total	Wash‐out group	Naïve group
**16‐5p**	195,392	219,327	193,101	334,792	360,419	258,042	214,719	254,695	140,517	463,540^a^	468,442	458,638
**22‐3p**	9702	9216	9702	20,805^b^	35,606	12,582	14,555	21,876	10,222	35,050^a^	36,587	33,514^a^
**30c‐5p**	1561	1438	1638	7517	6989	7517	2852^c^	3488	2547^c^	11,797	10,295	13,159
**140‐5p**	635	435	641	2528	2649	1696	1533	1816	1249	3590^a^	3246	6429
**340‐5p**	381	298	614	1353^b^	1476	1008	833	847	831	3333^a^	3415	3250
**494‐3p**	56	40	69	267	405	191	70	61	70	411	269	456

**^a^** t0 vs t6m ‐ *p* < 0.05 (Tukey Test).

**^b^** t0 vs t1m ‐ p < 0.05 (Tukey Test).

**^c^** t1 vs t3m ‐ p < 0.05 (Tukey Test).

**Figure 4:**
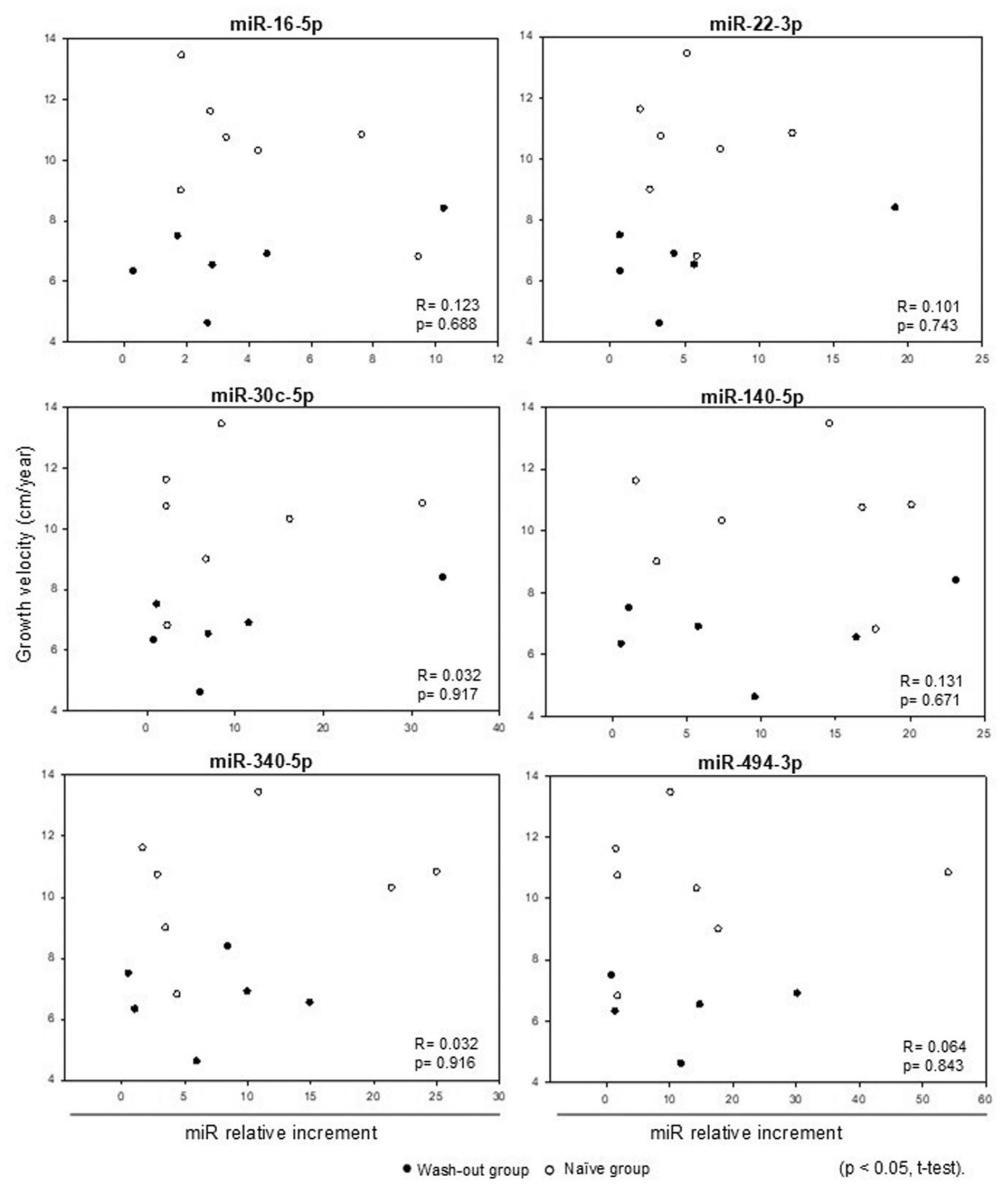
Comparison between growth velocity (cm/year) and the relative increment in miRNA levels: basal versus 6‐months timepoints of GH treatment.

**Figure 5:**
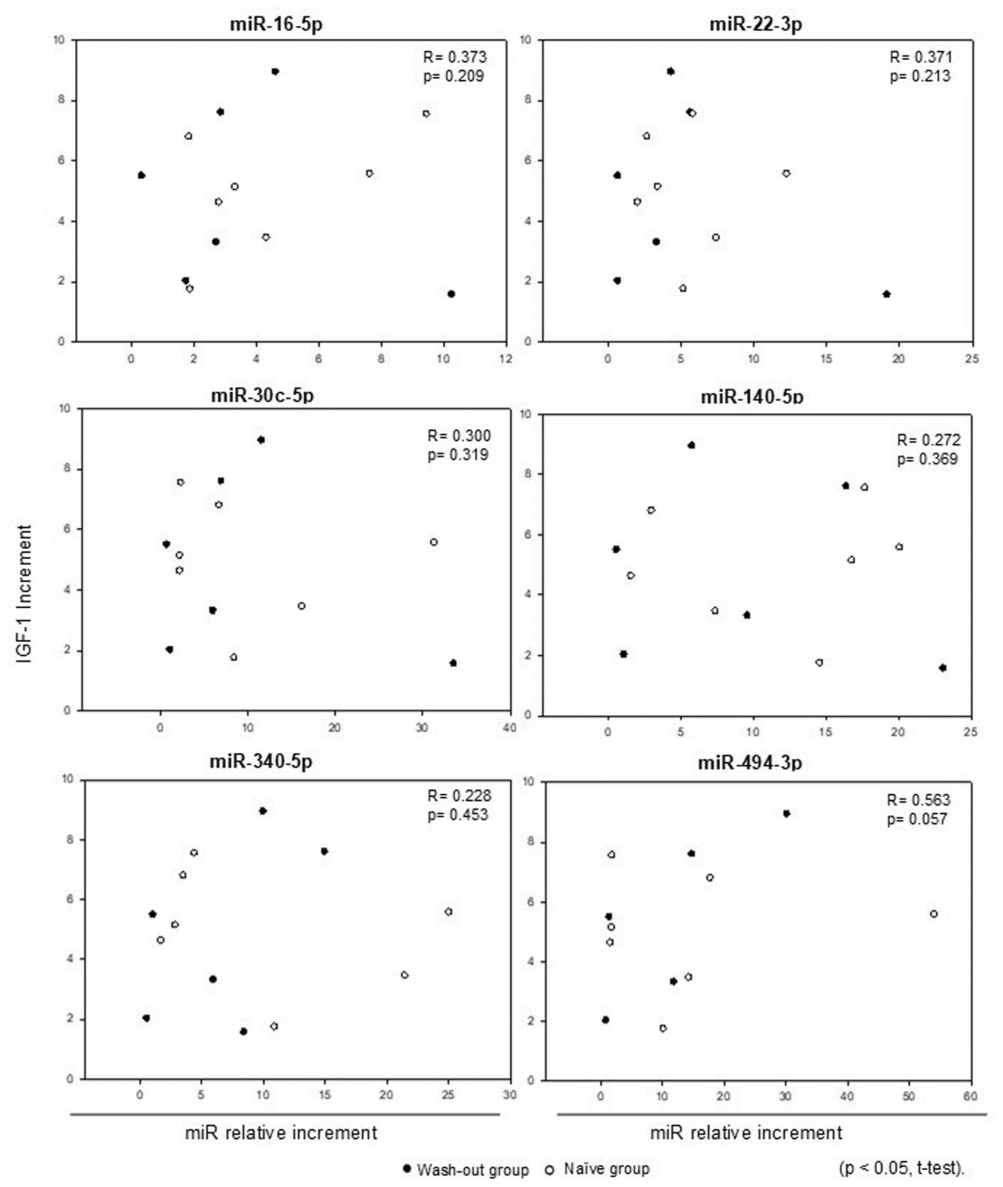
Comparison between the relative IGF‐1 increment and the relative increment in miRNA levels: basal versus 6‐months timepoints of GH treatment.

No significant correlation was identified between each miRNA increment and with annualized growth velocity (Figure 4) or (basal to 6‐month) with the relative increase in IGF‐1 levels (Figure 5). Additionally, when the analysis was performed separately within the wash‐out and naïve groups—correlating growth velocity and the relative increase in IGF‐1 levels—no statistically significant differences were observed.

Clinical and biochemical characteristics of patients included in each group is described in Table 3. No significant differences in miRNA expression were identified comparing these two groups. However, a significant difference in height‐SDS between groups was observed at baseline and after 6 months of treatment (*p* = 0.008, Mann‐Whitney Test), as expected the treatment‐naïve group have higher height‐SDS as a consequence of previous GH therapy. Even within the wash‐out and naïve groups, we observed variability in height‐SDS among individuals. Therefore, we divided the patients in each group according to the median height gain in an attempt to identify potential correlations. The median height gain in the wash‐out group was 0.09 SDS, while in the naïve group it was 0.48 SDS (Table 4); and still no significant difference was observed between wash‐out and treatment‐naïve groups regarding the incremental variation in miRNA concentration. To further investigate the response to GH treatment, we evaluated the IGF‐1 peak levels in each group, along with IGF‐1 increment, defined as the change in IGF‐1 levels from baseline to 6 months. These parameters were assessed according to subgroups based on height gain (above or below the median) within both the wash‐out and naïve groups. No statistically significant differences in IGF‐1 peak levels or IGF‐1 increment were observed between the subgroups in either group (Table 4). We also did not find any significant correlation between height increment SDS and miRNA concentration. Although, we would like to point out an interesting observation: patient number 12 (naïve group) exhibited the lowest growth velocity, demonstrated the greatest IGF‐1 response, yet showed the least responsive miRNA profile.

**Table 3:** Clinical and biochemical characteristics of naïve‐treatment patients and patients who underwent a wash‐out period of 30 days before basal sample collection.

Case	Gender	Age	Height	Height SDS	BMI SDS	IGF‐1	Bone Age	Age	Height	Height SDS	BMI SDS	IGF‐1	Age	Height	Height SDS	BMI SDS	IGF‐1	Age	Height	Height SDS	BMI SDS	GV	GV SDS	IGF‐1	Bone Age
		Basal						1 month					3 months					6 months							
**Wash‐out**																									
2	M	8.93	134	0.31	1.23	54.10	7	9.03	135.2	0.41	1.56	43.23	9.20	136.3	0.44	1.66	42.31	9.45	137.4	0.4	1.21	6.54	1.70	41.13	8
3	M	11	143.2	−0.03	2.77	11.52	9	11.20	144.3	0.01	3.00	53.45	11.37	144.9	−0.06	2.99	66.68	11.6	147	0.06	2.61	6.33	1.85	63.40	10
6	F	4.49	95	−2.45	−1.22	67.07	3	4.59	95.70	−2.42	0.52	31.18	4.75	97.1	−2.31	−0.14	20.17	5.14	98	−2.49	0.82	4.62	−1.78	22.27	4.1
8	M	10.41	134.4	−0.87	−0.42	53.31	8.5	10.53	135.00	−0.85	−0.04	32.49	10.6	n.a.	n.a.	n.a.	n,a,	10.88	137.3	−0.77	−0.15	6.9	2.74	47.68	9
9	M	7.14	108.4	−2.65	0.96	85.01	7	7.24	109.4	−2.55	1.40	36.16	7.41	110.2	−2.55	1.23	22.53	7.64	112.6	−2.31	1.76	8.40	3.35	13.36	8
10	M	7.88	108.5	−3.23	1.23	14.14	6.5	7.98	109	−3.21	1.74	41.92	8.25	111.6	−2.96	1.87	40.21	8.52	113.3	−2.86	1.87	7.5	2.65	28.69	7
**No wash‐out**																									
5	M	14.97	146	−2.92	0.82	1.95	13	15.14	147.7	−2.81	1.21	14.02	15.31	149.4	−2.7	0.73	17.29	15.66	152.2	−2.51	0.63	8.99	5.49	13.36	13.5
7	M	8.03	103.8	−4.18	−1.67	3.78	4	8.13	104.7	−4.08	−0.78	17.55	8.32	107.2	−3.78	−1.16	8.80	8.68	110.5	−3.46	−1.39	10.31	6.39	13.1	5.5
11	F	10	114.7	−3.75	−1.71	2.72	10.8	10.10	115.1	−3.77	−1.31	9.22	10.27	118	−3.46	−1.35	9.61	10.54	120.5	−3.3	−1.72	10.74	5.4	14.02	10.8
12	M	9.85	120.5	−2.61	1.93	2.99	7	10.15	121.5	−2.65	2.03	32.75	10.32	123.5	−2.46	1.94	31.44	10.57	125.4	−2.34	1.99	6.8	2.53	22.66	7.5
13	M	3.44	80.1	−4.9	−0.15	1.96	2.6	3.62	82.6	−4.51	−0.75	1.96	3.80	84	−4.38	−0.4	3.83	4.02	87.9	−3.7	−1.02	13.44	5.56	3.44	3
14	M	1.48	76.5	−2.07	1.29	3.31	1.5	1.67	77.4	−2.44	1.82	7.50	1.83	80.4	−1.9	1.0	14.14	2.08	83	−1.59	1.87	10.83	1.20	18.47	2
16	M	6.1	97	−3.94	0.37	1.99	5	6.27	99.8	−3.54	0.77	16.11	6.43	101.5	−3.34	0.83	12.06	6.66	103.5	−3.15	0.75	11.6	6.39	9.22	5.5

*Note:* Gender M: male/F: female; Age: years; Bone age, years; Height, cm; zHeight, SDS; zBMI, SDS; Growth velocity, cm/year; zGV: growth velocity, SDS; IGF‐1, nmol/L.

**Table 4:** Relative peak increment of miRNAs, IGF‐1 peak levels, and IGF‐1 increment, presented according to treatment groups (wash‐out vs. naïve) and stratified by height‐SDS gain.

	Wash‐out group			Naïve group		
miRNA (increment)	Height gain			Height gain		
	≥ median	< median	*p*	≥ median	< median	*p*
**16‐5p**	2.94	2.69	0.40	3.44	1.83	0.23
**22‐3p**	3.62	3.33	0.70	4.41	2.67	0.23
**30c‐5p**	11.55	4.51	0.40	6.06	2.19	0.63
**140‐5p**	5.47	9.57	1.00	4.91	3.61	0.86
**340‐5p**	9.99	3.69	0.70	5.28	2.84	0.23
**494‐3p**	15.51	3.19	0.80	6.48	1.79	0.86
**IGF‐1 Peak**	32.48	41.13	0.40	12.06	13.69	0.23
**IGF‐1 Increment**	2.20	5.50	0.70	4.05	6.80	0.11

*Note:* Height gain was defined as the change in height‐SDS.

In the wash‐out group, height gain ≥ median corresponds to SDS‐height gain > 0.09, and height gain < median corresponds to SDS‐height gain < 0.09.

In the naïve group, height gain ≥ median corresponds to SDS‐height gain > 0.48, and height gain < median corresponds to SDS‐height gain < 0.48.

miRNA and IGF‐1 increments were calculated based on the fold increase from baseline. IGF‐1 peak levels were calculated in nmol/L.

## 4 Discussion

To minimize confounding factors, this study focused on prepubertal subjects with growth hormone deficiency caused by middle line defect associated with ectopic pituitary gland. Aiming to establish a panel of miRNAs that could be able to identify GH responsiveness and to be used as biomarkers of GH sensitivity, we measured circulating growth plate interfering miRNAs before and during GH treatment. To the best of our knowledge, this is the first study to examine changes in circulating miRNA levels in response to growth hormone (GH) treatment using digital PCR (dPCR).

As reported, microRNAs play a crucial role in gene expression, and their levels change throughout life [18] and are influenced by body weight. Among the miRNAs selected for this study to compose the panel, miR‐22‐3p, miR‐30c‐5p, miR‐140‐5p, miR‐340‐5p, miR‐494‐3p, and miR‐16‐5p showed significant increase throughout the 6 months of GH therapy. In spite of this, miRNA increment was not correlated to the IGF‐1 increment levels, annualized growth velocity, or growth velocity SDS.

In our study we observed a typical pattern of miRNA increment during GH treatment, characterized by significant increase after 1‐month and 6‐month timepoints of GH treatment, but with transient reduction at 3‐month. The same pattern of transient reduction at 3‐month GH therapy was observed for all miRNAs included in the panel. We hypothesized that this phenomenon could be related to the presence of higher density of GH receptors in the hepatocytes of GH deficient patients, followed by downregulation of GH receptors after starting GH therapy, with further stabilization of hepatocyte GH receptor density after 6 months of treatment.

We identified a significant increase of miR‐494‐3p. In a previous report [10], miR‐494‐3p, miR‐335‐5p, and miR‐199a‐5p, were significantly upregulated at 3 months, and both their baseline circulating levels and the changes observed in the first 3 months contributed substantially to explaining growth at 12 months of treatment, significantly improving growth prediction based on baseline clinical features. MiR‐494‐3p has not yet been studied in the context of bone or growth plate development, but it has been reported to promote hyperactivation of the PI3K/AKT pathway [19], and known to regulate hypertrophic chondrocyte differentiation, play a role in endochondral bone growth, inducing osteoblast differentiation [20].

In rats, miR 22‐3p regulates both the resting and growth zones of cartilage, without pushing either cell population toward a more proliferative or hypertrophic phenotype. Instead, it increased the production of proteins characteristic of both regions [21]. Other miRNAs, such as miR‐1‐3p, miR‐22‐3p, miR‐30c‐5p, miR‐122‐5p, and miR‐133a‐3p were also implicated in the regulation of proteins involved in bone and skeletal development, cell division, and cell differentiation. MiR‐30c‐5p was is involved in bone, cartilage, osteoblast and osteoclast development, as well as collagen catabolism. MiR‐22‐3p and, to a lesser extent, miR‐30c‐5p are linked to vesicle‐related pathways [22].

We also identified a significant increase of miR‐140p from the baseline to after 6 months of treatment. Recent report has provided insights into how miR‐140 is regulated in the growth plate and how it, in turn, influences chondrocyte functions. Sox9, a key transcription factor for cartilage formation and chondrocyte differentiation, positively regulates miR‐140 expression [23]. Earlier studies identified several downstream targets of miR‐140 in chondrocytes, including aspartyl aminopeptidase [24], which modulates bone morphogenetic proteins, and ADAMTS5 [25] an enzyme involved in cartilage matrix turnover. More recently, genome‐wide screening has identified and experimentally validated many bona fide targets of miR‐140 in chondrocytes, including genes involved in Wnt signaling [26].

In our study we observed a significant increase of miR‐340‐5p after 1 and 6 months of treatment. MiR‐340‐5p has not yet been studied in the context of bone or growth plate development, but it has been shown to inhibit the PI3K/AKT signaling pathway. In osteosarcoma, RUSC1‐AS1 is overexpressed and is shown to directly binds to and inhibits miR‐340‐5p, activating the PI3K/AKT signaling pathway [27].

MiR‐16 is usually employed as a normalizing miRNA, but we recognized a significant increase in miR‐16 expression during GH treatment, preventing its use as a normalizing gene, but allowing its use as a study miRNA included in our panel. Interestingly, the promoter region for the *MIR‐15b/16‐2* genes was reported be negatively regulated after binding of the transcription factor STAT5a (Signal Transducer and Activator of Transcription 5a). Therefore, the activation of STAT5a is able to reduce the expression level of mature miR‐15 and miR‐16 [28]. The effects of STAT5b upon miR‐15 and miR‐16 were not reported. As STAT5b is a downstream effector of the GH‐activated GHR, this opens a potential coordinated regulation, allowing communication between the GH/GHR axis and miR‐15/16 expression via STAT5b.

One strength of this study is that it involved a homogeneous study population with confirmed GH deficiency, as previously noted. However, this population typically shows a strong and relatively uniform response to GH therapy, which may reduce the variability needed to detect correlations with predictive biomarkers such as circulating miRs. In contrast, non‐GHD children such as idiopathic short stature (ISS) display more heterogeneous responses to GH, and future studies in such populations may be better suited to uncover potential predictive relationships between miR profiles and treatment outcomes.

Besides that, we would like to highlight a point that may assist future investigations: the identification of additional normalizing miRNAs—both growth plate‐associated and unrelated—is necessary. Such normalization strategies may enhance the reliability and interpretability of circulating miRNA analyses.

In conclusion, the panel standardized in this study was able to recognize some of the potential miRNAs differentially expressed during the use of GH, but it is still not able to correlates with the variable pattern of growth response observed during GH therapy in prepubertal children with GHD. Future increase in sample size and the addition of new miRNAs to this panel may offer a clinically useful panel of miRNAs to recognize individual sensitivity to GH treatment.

## Disclosure

The authors have nothing to report.

## Conflicts of Interest

The authors declare no conflicts of interest.
